# FOXO1 regulates Th17 cell-mediated hepatocellular carcinoma recurrence after hepatic ischemia-reperfusion injury

**DOI:** 10.1038/s41419-023-05879-w

**Published:** 2023-06-17

**Authors:** Haozhen Ren, Yuyan Chen, Zhengyi Zhu, Jinkun Xia, Shujun Liu, Yingzhe Hu, Xueqian Qin, Lu Zhang, Yitao Ding, Senzhe Xia, Jinglin Wang

**Affiliations:** 1 Division of Hepatobiliary and Transplantation Surgery, Department of General Surgery, Nanjing Drum Tower Hospital Clinical College of Nanjing University of Chinese Medicine, Nanjing, Jiangsu Province China; 2 Division of Hepatobiliary and Transplantation Surgery, Department of General Surgery, Nanjing Drum Tower Hospital, Affiliated Hospital of Medical School, Nanjing University, Nanjing, 210008 China; 3 Division of Hepatobiliary and Transplantation Surgery, Department of General Surgery, Nanjing Drum Tower Hospital Clinical College of Jiangsu University, Nanjing, Jiangsu Province China

**Keywords:** Cancer, Cell biology

## Abstract

**Background:**

Hepatic ischemia-reperfusion injury (IRI) is considered as an effecting factor for hepatocellular carcinoma (HCC) recurrence. Th17/Treg cells are a pair of essential components in adaptive immune response in liver IRI, and forkhead box O1 (FOXO1) has the properties of maintaining the function and phenotype of immune cells. Herein, we illuminated the correlation and function between Th17/Treg cell balance and FOXO1 in IRI-induced HCC recurrence.

**Methods:**

RNA sequencing was performed on naive CD4+ T cells from normal and IRI model mice to identify relevant transcription factors. Western blotting, qRT-PCR, immunohistochemical staining, and flow cytometry were performed in IRI models to indicate the effect of FOXO1 on the polarization of Th17/Treg cells. Then, transwell assay of HCC cell migration and invasion, clone formation, wound healing assay, and Th17 cells adoptively transfer was utilized to assess the function of Th17 cells in IRI-induced HCC recurrence in vitro and in vivo.

**Results:**

Owning to the application of RNA sequencing, FOXO1 was screened and assumed to perform a significant function in hepatic IRI. The IRI model demonstrated that up-regulation of FOXO1 alleviated IR stress by attenuating inflammatory stress, maintaining microenvironment homeostasis, and reducing the polarization of Th17 cells. Mechanistically, Th17 cells accelerated IRI-induced HCC recurrence by shaping the hepatic pre-metastasis microenvironment, activating the EMT program, promoting cancer stemness and angiogenesis, while the upregulation of FOXO1 can stabilize the liver microenvironment homeostasis and alleviate the negative effects of Th17 cells. Moreover, the adoptive transfer of Th17 cells in vivo revealed its inducing function in IRI-induced HCC recurrence.

**Conclusions:**

These results indicated that FOXO1-Th17/Treg axis exerts a crucial role in IRI-mediated immunologic derangement and HCC recurrence, which could be a promising target for reducing the HCC recurrence after hepatectomy.

**Figure:**
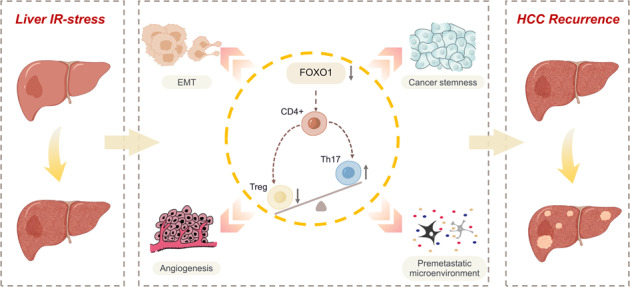
Liver IRI affects the balance of Th17/Treg cells by inhibiting the expression of FOXO1, and the increase of Th17 cells has the ability to induce HCC recurrence through EMT program, cancer stemness pathway, the formation of premetastatic microenvironment and angiogenesis.

## Introduction

Hepatectomy is the exact and effective treatment for early-stage hepatocellular carcinoma (HCC) [1, 2]. Hence, liver ischemia-reperfusion injury (IRI) has become an inevitable complication in clinical liver surgery [3]. Nevertheless, it has been confirmed that liver IRI stimulated by hepatic portal occlusion or liver transplantation is an independent risk factor for HCC recurrence after surgery [4]. Due to the reconstruction of the liver immune microenvironment and the destruction of the liver barrier caused by hepatic IR stress, the residual tumor cells are endowed with superior migration and invasion capabilities, which eventually progress to HCC recurrence [5, 6]. However, because of the complexity and variability of cellular and molecular components and targets that participated in liver IRI, the underlying mechanism of IRI-induced HCC recurrence has not been fully elaborated. Therefore, it is of great value to reveal promising targets and further explore the cellular and molecular mechanisms of IRI-induced HCC recurrence.

As a non-negligible impact, liver IRI can trigger inflammation outbreaks and derangement of the immune microenvironment. It was demonstrated that CD4+ T cells participated in liver IRI and performed a significant position in HCC tumor immunity [7, 8]. T helper type 17 (Th17) is a class of CD4+ T cells with special polarization characterized by the secretion of the IL-17A. Owing to the ability to promote the aggregation of neutrophils and induce the release of pro-inflammatory factors [9, 10], Th17 cells and related cytokines are classified as pro-inflammatory factors and have been proven to participate in hepatic IR stress [11, 12]. Meanwhile, it was indicated that Th17 cells have the ability to promote tumor growth, metastasis, invasion, and angiogenesis and are essential components of cancer immunity [13–15]. Regulatory T cells (Treg) are a class of CD4+ T cells with the ability to inhibit the inflammatory response, which is considered to be a pair of lymphocytes with opposite functions to Th17 cells [16]. As a pivotal member of the FOXO family, forkhead box O1 (FOXO1) exerts an irreplaceable role in maintaining the formation and differentiation of immune cells [17, 18]. It has demonstrated that FOXO1 can mediate the fate of Th17 cells and has the property of inhibiting the polarization and function of Th17 cells to inhibit the spread of inflammation [19–21]. However, the correlation between the increase of Th17 cell polarization stimulated by liver IRI and HCC recurrence and the regulatory function of FOXO1 in this process remains to be explored.

Herein, we revealed the correlation between FOXO1 and Th17/Treg cell balance and expounded the effect of Th17 cells in IRI-induced HCC recurrence. Through the detection of clinical and mice IRI models, it was demonstrated that after IR stress, hepatic inflammation response erupted, and the proportion of Th17/Treg cells was disordered. Besides, FOXO1 has the property to participate in the process of liver IRI by regulating the balance of Th17/Treg cells. Moreover, it was proved that Th17 cells have the ability to exacerbate HCC recurrence by promoting epithelial–mesenchymal transformation (EMT), cancer stemness, and the formation of a pre-metastasis microenvironment. Further, in order to illustrate the effect of Th17 cells in IRI-induced HCC recurrence, Th17 cells adoptively transferring experiment in vivo was also performed. In conclusion, this paper confirmed the correlation between FOXO1 and polarization of Th17/Treg cells and elaborated on the effect of Th17 cells in IRI-induced HCC recurrence, providing a novel target and direction for basic and clinical research.

## Materials and methods

### Animals

Animal experiments were performed on male 6–8 weeks C57BL/6J mice, which were purchased from the Experimental Animal Center of Drum Tower Hospital, Nanjing University of Medical School, and fed in a specific sterile environment. All animal experiments were approved by the Institutional Animal Care and Use Committee of Nanjing University in accordance with the National Institutes of Health (NIH) Laboratory Animal Care and Use Guidelines (Nanjing, China; no. 2020AE01042). All mice were randomly assigned to the experiments.

### Human liver IRI samples

The human liver samples were acquired from patients with benign lesions undergoing hepatectomy in Nanjing Drum Tower Hospital. The clinical diagnosis of these patients included focal nodular hyperplasia, hemangioma, hepatolithiasis, and angiomyolipoma, without a history of autoimmune disorders, hepatitis, malignancy, immune deficiencies, or HIV infection. Liver samples of HCC recurrence were obtained from patients with recurrent HCC. All specimen acquisitions were communicated with patients, and signed informed consent. All human studies were supported by the Human Ethics Committee of Nanjing Drum Tower Hospital following the Declaration of Helsinki (Nanjing, China; no. 2019-257-02).

### Mice hepatic IRI and HCC recurrence model

In 4–8 weeks male C57BL/6 mice, the mice IRI model was constructed by continuously clamping the portal vein for 90 min and then reperfusion for 6 h. The mice were sacrificed after anesthesia by isoflurane, and serum and liver samples were collected for determination. The mice model of FOXO1 upregulation was established by continuous intraperitoneal injection of Resveratrol (Res; 501–36–0; Selleck Chem, 25 mg/kg i.p.) for 14 days. The HCC recurrence models were constructed by injecting 2 × 10^6^ Hepa1–6 cells into the portal vein. And mice were sacrificed under anesthesia by isoflurane after 4 weeks. The liver tissues were collected for testing. The mice in different groups were randomly numbered on the tail, and hepatic injury was assessed using Suzuki’s criteria by five different pathologists who were blind to groups.

### Liver colonization model of HCC cells

The mice IRI model was constructed as described above, and 2 × 10^6^ GFP+Hepa1–6 cells were injected into the portal vein after liver ischemia for 90 min to simulate residual HCC cells, and liver tissue was collected on the 1st and 3rd day after the operation, the colonization of GFP+Hepa1–6 cells in the liver was detected by flow cytometry.

### Detection of liver enzymes in serum

Mice in each group were anesthetized by isoflurane, and peripheral blood samples were obtained through eyeball blood collection. The levels of serum liver enzymes, including alanine aminotransferase (ALT), and aspartate aminotransferase (AST), were determined by a biochemical analyzer (Fuji, Tokyo, Japan).

### Real-time quantitative polymerase chain reaction

Extraction of RNA from human and mouse liver tissues by Trizol reagent (Vazyme Biotech, Nanjing, China). The concentration and purity of the extracted total RNA were detected by spectrophotometer (Thermo Fisher Scientific, Waltham, USA), and record the concentration and purity of RNA. RNA with OD260/OD280 in the range of 1.8–2.0 can be used for subsequent detection. And Superscript II Reverse Transcriptase kit (Vazyme Biotech, Nanjing, China) was used to achieve RNA Reverse transcription into cDNA on a ProFlex™ 2×Flat PCR (Applied Biosystems, Foster City, CA, USA). And the expression level of target mRNA was quantitatively detected by SYBR Green PCR Master Mix (Vazyme Biotech, Nanjing, China) on an ABI PRISM 7500 Real-Time PCR System (Applied Biosystems, Foster City, CA, USA). β-actin was used as a housekeeping gene to quantify the expression of target genes by 2^−△△CT^. The list of primers required for the entire process is presented in Supplementary Table S2.

### Western blotting assay

Protein was extracted from samples by RIPA Lysis Buffer (Thermo Fisher Scientific, Waltham, USA), and BCA assay (Thermo Fisher Scientific, Waltham, USA) was utilized indicated protein concentration. An equal amount of protein was loaded on SDS-PAGE, transferred, and incubated with primary antibodies (Supplemental Table S1). After incubation with a secondary antibody, images of corresponding bands were obtained using the ECL chemiluminescence imaging system and exposed to on X-ray film (Kodak, Japan). The grayscale value of the image is quantified by Image J, and calculate the respective grayscale value ratio of bands representing objective proteins to a band of β-actin to acquire the variation tendency of each protein.

### Extraction of intrahepatic lymphocytes

Mice were anesthetized by inhaling isoflurane, the portal of the mice was exposed, and a soft catheter was inserted into the portal vein of the mice. The prewarmed Hank’s balanced salt solution (Sigma-Aldrich, MO, USA) was pumped into the liver to remove residual blood. And the livers were cut up and digested in type IV collagenase (BS165, Biosharp, Hefei, China) for 1 h and ground in a 70 μm cell strainer (BS-70-CS, Biosharp, Hefei, China) to obtain a cell suspension, and the cells were subsequently centrifuged at 700*g* for 5 min at 4 °C. After centrifugation, cell suspension was re-suspended with 40% percoll (BS909, Biosharp, Hefei, China) solution and slowly added to 70% percoll solution for gradient centrifugation to separate lymphocytes. The collected lymphocytes are washed and centrifuged for subsequent detection.

### Lymphocyte phenotype detected by flow cytometry

Intrahepatic lymphocytes were extracted by the above method. The following antibodies that bind to surface or intracellular markers of mice lymphocytes were used: FITC anti-mouse CD4 (clone H129.19, Biolegend, San Diego, CA, USA), APC anti-mouse CD25 (clone 3C7, Biolegend), PE anti-mouse FOXP3 (clone MF-14, Biolegend), PE anti-mouse IL-17A (clone TC11–18H10.1, Biolegend). FITC-CD4 and APC-CD25 were used to indicate Treg cell surface markers, and PE-FOXP3 was used to indicate intracellular markers in Treg cells. FITC-CD4 was used to indicate surface markers in Th17 cells, and PE-IL-17A was used to indicate intracellular markers in Th17 cells. Wash cells after flow cytometry antibody staining in the dark and resuspend with 100 µL staining buffer for flow cytometry. The results were captured by a BD FACS Aria III Flow Cytometer (BD Bioscience, San Jose, CA, USA) and analyzed using FlowJo 10.4 software (BD Bioscience, San Jose, CA, USA).

### Extraction and differentiation of Th17 cells in vitro

Naive CD4+ T cells from WT mice were extracted by magnetic bead negative selection using the EasySep™ mouse naive CD4+ T cell isolation kit (Catalog # 19765, Stem Cell Technology, Vancouver, Canada). Extracted Naive CD4+ T cells were cultured in 10% FBS + RPMI 1640 medium with 100 U/mL of penicillin and streptomycin and induced in plates cultured with 2 mg/mL anti-CD3 (05112–25–100, Biogems, NJ, USA) and 2 mg/mL anti-CD28 (10312–25–100, Biogems). The conditions for Th17 cell polarization in vitro: IL-6 (10 ng/mL, 78052, Stem Cell Technology), TGF-β1 (5 ng/mL, 100-21C-10, PeproTech, NJ, USA), IL-23 (25 ng/mL, 200-23-10, PeproTech), IL-1β (10 ng/mL, 78035, Stem Cell Technology). After naive CD4+ T cells were stimulated, cells were collected, and Th17 cells were quantified by flow cytometry.

### Th17 cells adoptively transfer in vivo

Naive CD4+ T cells were extracted from WT mice and cultured for 5 days under the conditions of Th17 cell polarization in vitro. The above mice IRI-induced HCC recurrence model was constructed and adoptively transferred Th17 cells into the HCC recurrence model via splenic injection (2 × 10^5^ cells). And mice were sacrificed under anesthesia by isoflurane after 4 weeks. The liver tissues were collected for testing.

### Hematoxylin-eosin staining and Immunohistochemical staining

Liver sections from humans and mice were fixed with paraformaldehyde and processed into wax blocks. Hematoxylin and eosin (H&E) staining were utilized to observe liver histopathological changes. and liver damage degree was quantified by Suzuki’s criteria. For immunohistochemical staining, the following antibodies are required: FOXO1 antibody (1:300, Ab52857, Abcam), IL-17A antibody (1:300, PA5-79470, Invitrogen), FOXP3 antibody (1:300, Ab215206, Abcam). Images were captured by Leica microscope.

### Cell culture

LM3 (human HCC cell line), Huh7 (human HCC cell line), Hepa1–6 (Mice HCC cell line), and HUVECs (Human Umbilical Vein Endothelial Cells) cell lines were purchased from ATCC (Manassas, VA, USA). LM3, Huh7, and Hepa1–6 cells were cultured in DMEM medium mixed with 10% FBS. HUVECs were cultured in DMEM/F12 medium mixed with 10% FBS. Mycoplasma contamination was conducted by mycoplasma test kit (4460623, Thermo Fisher Scientific, Waltham, USA) every 3 months for all cells and consistently tested negative.

### Co-culture system

Th17 cells were polarized and co-cultured with HCC cells (LM3 and Huh-7) and HUVECs. HCC cells or HUVECs were seeded at 4 × 10^5^ cells/well in the lower chamber of the Transwell plate (Corning, USA), while Th17 cells were plated in the upper chambers at 2 × 10^5^ cells/well. After incubation for 48 h, collect the lower layer cells for further analysis.

### Transcriptome sequencing analysis

Naive CD4+ T cells from wide type (WT) mice were extracted by magnetic bead negative selection using the EasySep™ mouse naive CD4+ T cell isolation kit (Catalog # 19765, Stem Cell Technology, Vancouver, Canada). Extracted Naïve CD4+ T cells were stored in dry ice and sent to OE Biotech Co., Ltd. for transcriptome sequencing. Total RNA was extracted using the mirVana miRNA Isolation Kit (Ambion) following the manufacturer’s protocol. RNA integrity was evaluated using the Agilent 2100 Bioanalyzer (Agilent Technologies, Santa Clara, CA, USA). The samples with RNA Integrity Number ≥ 7 were subjected to the subsequent analysis. The libraries were constructed using TruSeq Stranded mRNA LTSample Prep Kit (Illumina, San Diego, CA, USA) according to the manufacturer’s instructions. Then these libraries were sequenced on the Illumina sequencing platform (HiSeqTM 2500 or Illumina HiSeq X Ten), and 125 bp/150 bp paired-end reads were generated. FPKM value of each gene was calculated using cufflinks, and the read counts of each gene were obtained by htseq-count. DEGs were identified using the DESeq R package functions estimateSizeFactors and nbinomTest. *p* Value < 0.05 and fold change > 2 or fold change < 0.5 was set as the threshold for significantly differential expression. Hierarchical cluster analysis of DEGs was performed to explore gene expression patterns. GSEA enrichment analysis of DEGs was respectively performed using R based on the hypergeometric distribution. The original RNA-seq data have been reported to the Sequence Read Archive (PRJNA938812).

### Statistical analysis

All experimental data were systematically analyzed by GraphPad Prism software. All data are expressed as the means ± SEM. All data were first tested for normality via the Kolmogorov–Smirnov test. Normally distributed data were compared by Student’s *t*-test. Otherwise, the Wilcoxon rank-sum test was applied. A one-way analysis of variance was adopted to compare differences among multiple groups. Flow cytometry data were processed using FlowJo (BD Bioscience, San Jose, CA, USA).

## Results

### Hepatic IRI initiates liver damage

**Fig. 1:**
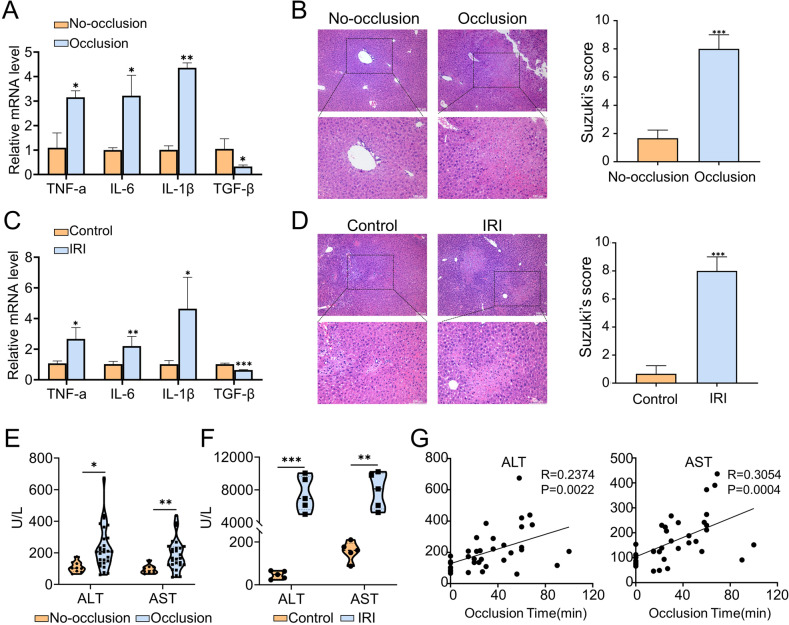
Hepatic IRI initiates liver damage. **A** The mRNA levels of TNF-a, IL-6, IL-1β, and TGF-β in clinical samples (*n* = 5 per group). **B** Liver representative H&E staining in clinical samples (*n* = 5 per group). **C** The mRNA levels of TNF-a, IL-6, IL-1β, and TGF-β in mice samples (*n* = 5 per group). **D** Liver representative H&E staining in mice samples. Scale bars, 100 μm (*n* = 5 per group). **E** The levels of serum ALT and AST in clinical samples. **F** The levels of serum ALT and AST in mice serum (*n* = 5 per group). **G** Correlation between cumulative blocking time and postoperative serum ALT/AST levels, *n* = 37. mean ± SEM, *p < 0.05, **p < 0.01, ***p < 0.001, Student *t*-test.

The groups of clinical samples were divided into a no-occlusion group and an occlusion group according to whether the hepatic portal block and the expression of inflammatory factors, liver function, and histological changes were assessed. It was illustrated that the inflammatory makers, including TNF-a, IL-6, IL-1β, and TGF-β, were dramatically altered after the liver undergoes IR-stress at the mRNA level (Fig. 1A). As shown in Fig. 1B, compared to a no-occlusion group, the histopathological features of occlusion group presented remarkable necrosis, vacuole, and congestion. Moreover, it was intuitively illustrated that the level of serum ALT/AST were increased dramatically after hepatic portal occlusion (Fig. 1E) and was positively correlated with the cumulative occlusion time (Fig. 1G). In addition, mice IRI models were established to validate the changes initiated by IR stress. Consistent with the results of clinical samples, gene expression of pro-inflammatory factors is increased, while the expression of anti-inflammatory factors is relatively decreased (Fig. 1C), the histological manifestations were severely damaged (Fig. 1D), and the serum ALT/AST also showed the same upward trend (Fig. 1F). These results indicated that hepatic IRI could trigger the activation of hepatic inflammation and induce severe damage.

### IR-stress induces the imbalance of the Th17/Treg cells ratio

**Fig. 2:**
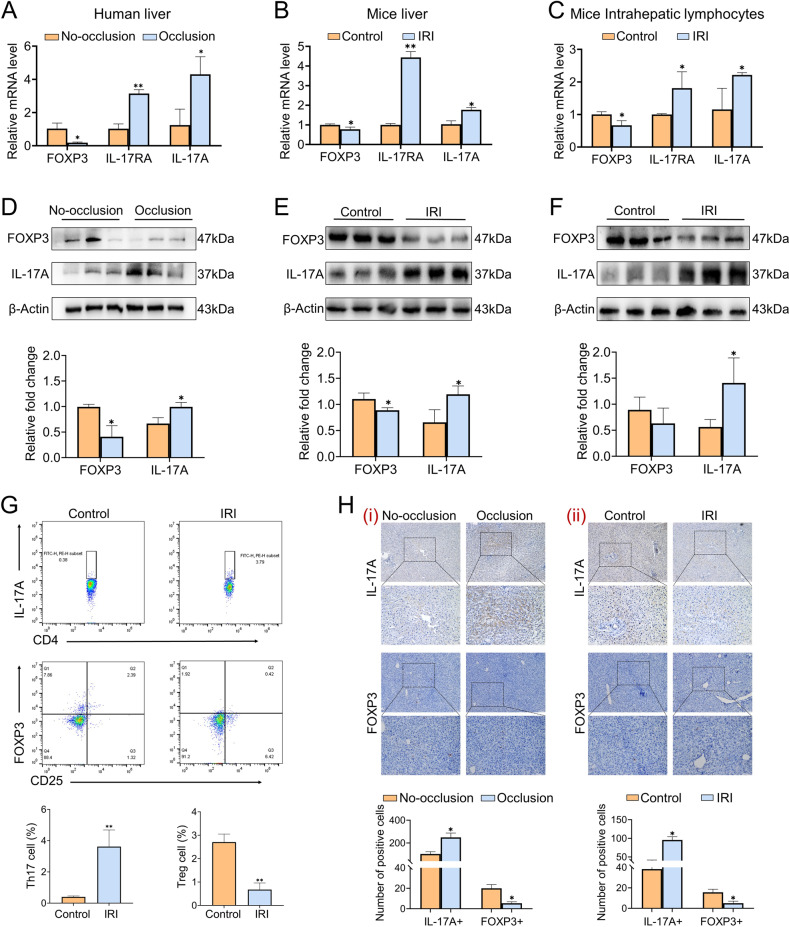
The imbalance of Th17/Treg cells after liver IRI. **A** The mRNA levels of FOXP3, IL-17RA, and IL-17A in clinical liver samples (*n* = 3 per group). **B** The mRNA levels of FOXP3, IL-17RA, and IL-17A in mice liver samples (*n* = 3 per group). **C** The mRNA levels of FOXP3, IL-17RA, and IL-17A in mice intrahepatic lymphocytes (*n* = 3 per group). **D** Western blotting analysis of FOXP3, IL-17A in clinical liver samples (*n* = 3 per group). **E** Western blotting analysis of FOXP3, IL-17A in mice liver samples (*n* = 3 per group). **F** Western blotting analysis of FOXP3, IL-17A in mice intrahepatic lymphocytes (*n* = 3 per group). **G** Identification and quantification of intrahepatic lymphocytes phenotype transition by flow cytometry (*n* = 3 per group). **H** Immunohistochemistry staining and quantification of FOXP3 and IL-17A in human and mice liver. Scale bars, 100 μm (*n* = 4–5 per group). mean ± SEM, *p < 0.05, **p < 0.01, Student *t*-test.

There have been elucidated the function of CD4+ T cells in IR-induced immune and inflammatory cascades [22**–**24]. Th17/Treg cells are a pair of the essential subtypes of CD4+ T cells, which are currently considered as a pair of lymphocytes with opposite effects, exerting an indispensable role in inflammation and immune response [25, 26]. In liver IRI models, it was indicated that Th17 cells-related factors and Treg cells-related factors presented opposite expression trends at the mRNA (Fig. 2A, B) and protein (Fig. 2D, E) levels both in clinical and mice liver samples. Furthermore, the same expression trends of Th17/Treg cell-related factors under liver IR stress were identified in mice hepatic lymphocytes (Fig. 2C, F). In order to illustrate differential expression trends of Th17 cells and Treg cells intuitively, flow cytometry (Fig. 2G) and immunohistochemistry staining (Fig. 2H) was utilized to demonstrate the alteration in the ratio of Th17/Treg cells. These results implied that the hepatic immune microenvironment was more inclined towards the polarization of Th17 cells after ischemic stress, while the polarization of Treg cells characterized as anti-inflammatory effects were inhibited.

### FOXO1 participates in the process of liver IRI

**Fig. 3:**
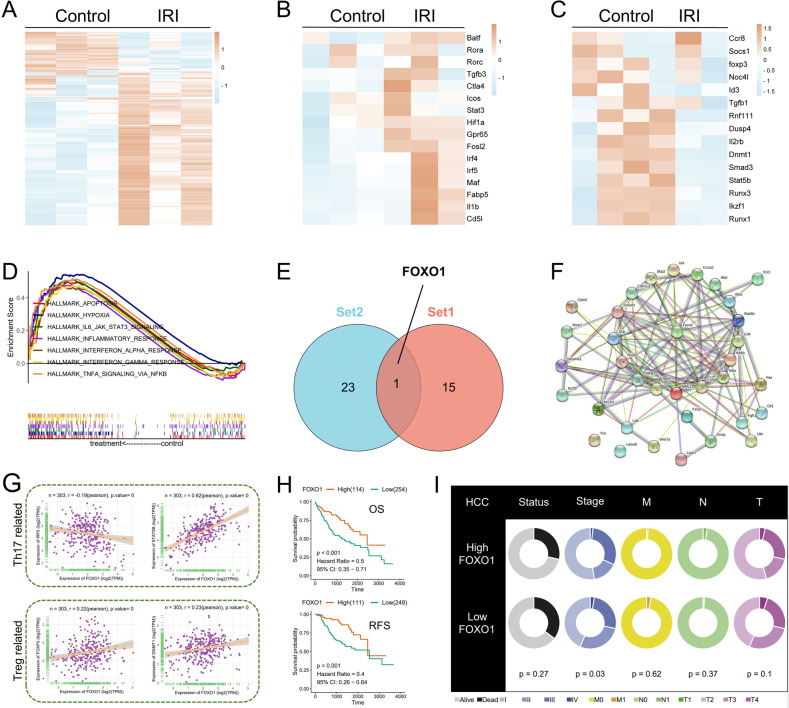
Transcriptome analysis of naive CD4+ T cells and the exploration of FOXO1 in the database. **A** Heat map of DEGs in naive CD4+ T cells with or without liver IRI (*n* = 3 per group). **B** Heat map of Th17 cells related genes in DEGs. **C** Heat map of Treg-related genes in DEGs. **D** DEGs enrichment signaling pathway. **E** Venn diagram representing the intersection of two sets of DEGs-related transcription factors. **F** Analysis of protein-protein relationships between FOXO1 and the genes on the Th17 cells differentiation pathway by STRING database. **G** Correlations between FOXO1 and IRF5, STAT5B, FOXP3, and DNMT1. **H** OS and RFS were determined to study the significance of FOXO1 on HCC patients. **I** Clinical information on FOXO1.

To further explore the specific mechanism of liver immune microenvironment disorder initiated by liver IRI, RNA sequencing was performed on naive CD4+ T cells from mice with or without IRI. The significant alterations in the gene expression profile of naive CD4+ T cells have been observed by heatmaps (Fig. 3A). Moreover, the differentially expressed genes (DEGs) specifically participated in the activity, polarization, and function of T cells were analyzed. Generally, the DEGs related to T cell activity favored Th17 cell programs over Treg cell programs (Fig. 3B). Genes positively regulate Th17 cells differentiation and function, including Fabp5 [27] and Fosl2 [28], were significantly upregulated. Conversely, the expression of related genes necessary for Treg cell activity tended to be suppressed (Fig. 3C).

Under the background of liver IRI, it was indicated that most of the DEGs are enriched in inflammation-related pathways and apoptosis pathways (Fig. 3D). In order to discover the underlying regulatory mechanism, transcription factor correlation analysis was conducted on the DEGs. Surprisingly, through transcription factor correlation analysis, FOXO1 was screened out as the intersection of the two sets and considered to be involved in the regulation of DEGs (Fig. 3E). This means that the FOXO1 might exert an essential function in liver IRI and participate in regulating the changes of the hepatic microenvironment. In addition, the protein–protein interaction network map revealed the tight correlation between FOXO1 and the genes on the Th17 cells differentiation pathway (Fig. 3F). In order to further explore the correlation between FOXO1 and polarization of lymphocytes, the preliminary exploration of the database illustrated that FOXO1 was negatively correlated with the Th17 cells positive regulator gene IRF5 [29], and positively correlated with the Th17 cell negative regulator gene STAT5B [30], while positively correlated with Treg cells-related genes FOXP3 [31] and DNMT1 [32] (Fig. 3G). According to the clinical information of HCC patients included in the database, the patients with the high expression of FOXO1 had both longer overall survival (OS) and recurrence-free survival (Fig. 3H). Moreover, FOXO1 expression was not significantly associated with individual pathological TNM stage and clinical status but was a momentous prognostic indicator for the overall clinical stage (Fig. 3I).

### The expression trend of FOXO1 after liver IR-stress

**Fig. 4:**
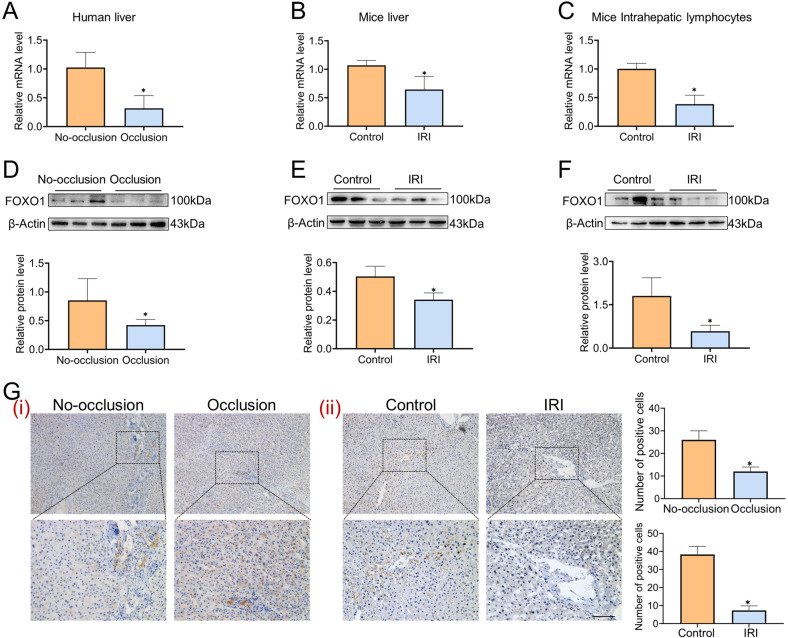
FOXO1 is downregulated after liver IRI. **A** The mRNA levels of FOXO1 in clinical liver samples (*n* =3 per group). **B** The mRNA levels of FOXO1 in mice liver samples (*n* = 3 per group). **C** The mRNA levels of FOXO1 in mice hepatic lymphocytes (*n* = 3 per group). **D** Western blotting analysis of FOXO1 in clinical liver samples (*n* = 3 per group). **E** Western blotting analysis of FOXO1 in mice liver samples (*n* = 3 per group). **F** Western blotting analysis of FOXO1 in mice intrahepatic lymphocyte (*n* = 3 per group). **G** Immunohistochemistry staining and quantification of FOXO1 in human (i) and mice (ii) liver (*n* = 4–5 per group). Scale bars, 100 μm. mean ± SEM, *p < 0.05, Student *t*-test.

The crucial role of FOXO1 in the regulation of hepatic IRI has been verified, and the next step is to confirm the expression trend of FOXO1 in IRI models. It was shown that FOXO1 presented a decreasing trend at the mRNA and protein levels in clinical (Fig. 4A, D) and mice (Fig. 4B, E) liver samples, and the same decreased trend was observed in hepatic lymphocytes (Fig. 4C, F). And immunohistochemical images confirmed the expression trend of FOXO1 (Fig. 4G). These results comprehensively indicated that FOXO1 expression was inhibited under hepatic IRI.

### FOXO1 can ameliorate inflammation response and disturbance of Th17/Treg cells

**Fig. 5:**
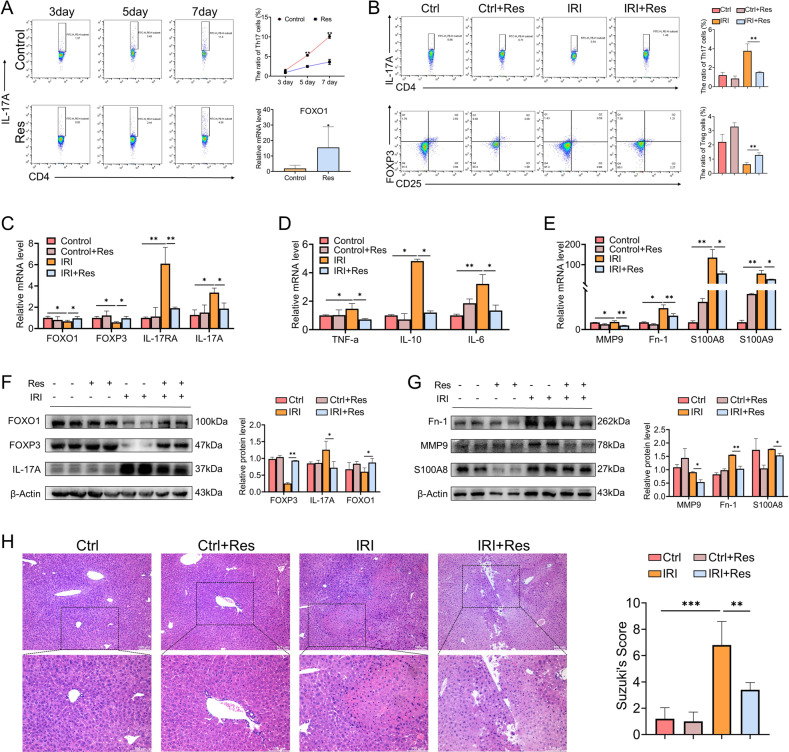
Upregulated FOXO1 expression can ameliorate inflammation response and disturbance of Th17/Treg cells. **A** Identification and quantification of Th17 cell proportion by flow cytometry after upregulation of FOXO1 expression in vitro and the mRNA levels of FOXO1 in lymphocytes were validated by qPCR (*n* = 3 per group). **B** Identification and quantification of Th17/Treg cells proportion by flow cytometry after up-regulation of FOXO1 expression in mice model (*n* = 3 per group). **C**–**E** The mRNA levels of FOXO1, TNF-a, IL-10, IL-6, FOXP3, IL-17RA, IL-17A, MMP9, Fn-1, S100A8, S100A9 in mice liver after upregulation of FOXO1 expression (*n* = 3 per group). **F**, **G** Western blotting analysis of FOXO1, FOXP3, IL-17A, MMP9, Fn-1, S100A8 after upregulation of FOXO1 expression in each group. **H** Liver representative H&E staining in mice liver (*n* = 4–5 per group). Scale bars, 100 μm. mean ± SEM, *p < 0.05, **p < 0.01, ***p < 0.001, Student *t*-test.

In order to illuminate the effect of FOXO1 in Th17 cells, lymphocytes were extracted and cultured under Th17 cells polarized conditions in vitro, and intracellular FOXO1 expression was up-regulated by the addition of Res and verified the FOXO1 expression level by qPCR. Compared with the control group, it was indicated that the polarization of Th17 cells in the Res group was blocked partially (Fig. 5A). This result demonstrated that at the cellular level, up-regulation of FOXO1 has the ability to inhibit the polarization of Th17 cells.

Besides, the function of FOXO1 was further explored in vivo. Compared with control groups, mice with up-regulated FOXO1 were able to partially reduce the increased polarization of Th17 cells induced by hepatic IRI and simultaneously induced the polarization of Treg cells (Fig. 5B). And the reversal of Th17/Treg cells ratio disorder was also reflected in the mRNA (Fig. 5C) and protein (Fig. 5F) levels of related factors, suggesting that the immune microenvironment tended to Th17 cells polarization could be reshaped by the upregulation of FOXO1. In addition, the mRNA level of liver inflammatory factors (Fig. 5D) and H&E staining (Fig. 5H) were detected to confirm that FOXO1 exerts a prominent role in attenuating IR-induced inflammatory damage. Due to the characteristics of participating in the recurrence and metastasis of a variety of cancers, Fn-1, S100A8, S100A9, and MMP9 were regarded as the main components of the formation of the pre-metastatic microenvironment. Nevertheless, it was demonstrated that these pre-metastatic microenvironment-related factors participated in the construction of the chaotic microenvironment after liver IRI, while the up-regulation of FOXO1 could attenuate the expression of these factors and stabilize the hepatic microenvironment (Fig. 5E, G). These results indicated that FOXO1 has a feature to alleviate the inflammatory response caused by IR stress, ameliorate the formation of the pre-metastatic microenvironment, and suppress the polarization of Th17 cells both in vivo and in vitro.

### Upregulation of FOXO1 can reduce HCC recurrence

**Fig. 6:**
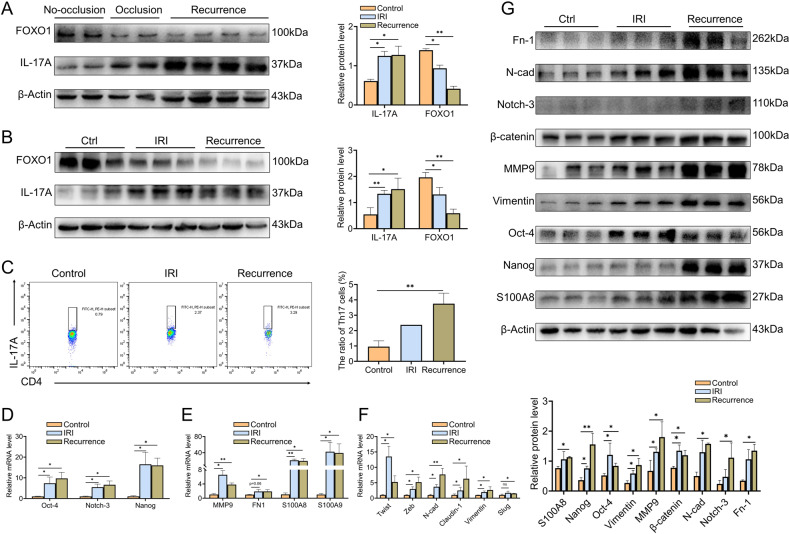
The changes of the microenvironment in the liver with IRI and HCC recurrence. **A** Western blotting analysis of FOXO1, IL-17A in human samples. **B** Western blotting analysis of FOXO1, IL-17A in mice samples (*n* = 3 per group). **C** Identification and quantification of Th17 cell proportion by flow cytometry in mice liver samples. **D**–**F** The mRNA levels of cancer stemness indicators (**D**), pre-metastasis microenvironment indicators (**E**), and EMT indicators (**F**) in mice samples (*n* = 3 per group). **G** Western blotting analysis of EMT indicators, cancer stemness indicators, and pre-metastasis microenvironment indicators in mice samples (*n* = 3 per group). Mean ± SEM, *p < 0.05, **p < 0.01, Student *t*-test.

Furthermore, the relationship between hepatic IR stress and HCC recurrence was further explored. Coincidentally, it was illustrated that the expression of FOXO1 presented a downward trend (Fig. 6A, B), whereas the expression of IL-17A and the infiltration abundance of Th17 cells were increased in the HCC recurrence model (Fig. 6C). In addition, the indicators of EMT, cancer stemness, and pre-metastatic microenvironment-related factors were the rise in the livers with IRI or HCC recurrence (Fig. 6D–G). Therefore, we assessed that there is a certain correlation between liver IRI and HCC recurrence, and Th17 cells and FOXO1 might have participated in HCC recurrence.

**Fig. 7:**
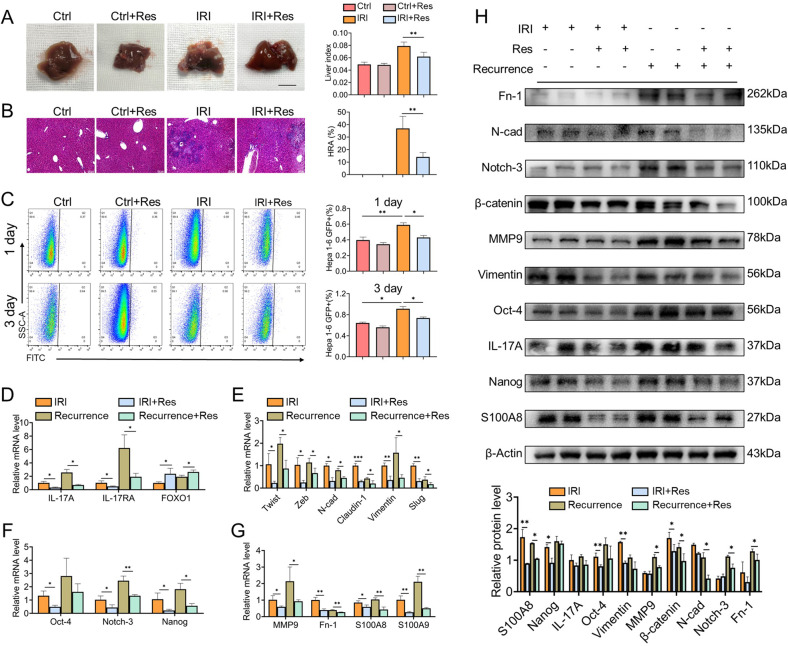
Upregulation of FOXO1 ameliorates the microenvironment in liver IRI and reduces HCC recurrence. **A** Liver tissue images of mice with HCC recurrence and liver index (liver weight/body weight), *n* = 4–5, Scale bars, 1 cm. **B** H&E staining to observe the morphology of HCC recurrence and hepatic replacement area (HRA) of HCC recurrence, *n* = 4–5. **C** Flow cytometric analysis and quantification of the colonization ratio of GFP+ Hepa1–6 cells in mice liver 1 and 3 days, *n* = 3. **D**–**G** The mRNA levels of FOXO1 and Th17 cell-related factors IL-17A, IL-17RA (**D**), EMT indicators (**E**), cancer stemness indicators (**F**), and pre-metastasis microenvironment indicators (**G**) in each group (*n* = 3 per group). **H** Western blotting analysis of EMT indicators, cancer stemness indicators, and pre-metastasis microenvironment indicators in each group. Mean ± SEM, *p < 0.05, **p < 0.01, ***p < 0.001, Student *t*-test.

To further reveal the correlation between liver IRI and HCC recurrence and the function of FOXO1 in HCC recurrence, the IRI-induced HCC recurrence models were established for detection. According to the condition of tumorigenesis in the gross liver (Fig. 7A) and microscopic recurrence by H&E staining (Fig. 7B), it was illuminated that liver IRI was a predisposing factor for HCC recurrence, while the upregulation of FOXO1 can partially alleviate HCC recurrence. Moreover, in order to assess the attractiveness of the hepatic microenvironment for residual HCC cells, GFP+ Hepa1-6 cells were injected into the portal vein to simulate residual HCC cells after hepatectomy. As shown in Fig. 7C, the colonization rate of GFP+ Hepa1-6 cells in the liver increased significantly after IRI, while the colonization rate was decreased after the upregulation of FOXO1, suggesting that the upregulation of FOXO1 can attenuate the colonization of residual HCC cells in the liver and reduce the susceptibility of the hepatic microenvironment to recurrence. Besides, upregulation of FOXO1 could reduce the expression of IL-17A and IL-17RA in the liver immune microenvironment constructed by IRI and HCC recurrence, and inhibit the activation of the EMT program, cancer stemness and the formation of the pre-metastatic microenvironment at mRNA levels, thereby ameliorating the hepatic microenvironment (Fig. 7D–G). Besides, the changes in protein expression level were shown in Fig. 7H.

These results verified that the EMT program, cancer stemness, and pre-metastatic microenvironment-related factors might be involved in the formation of IRI-induced disorganized liver microenvironment, which could attract the colonization of HCC cells and eventually contribute to HCC recurrence, while FOXO1 had a function to stabilize the hepatic microenvironment and alleviate the negative effects of IRI.

### The function of Th17 cells in HCC recurrence

**Fig. 8:**
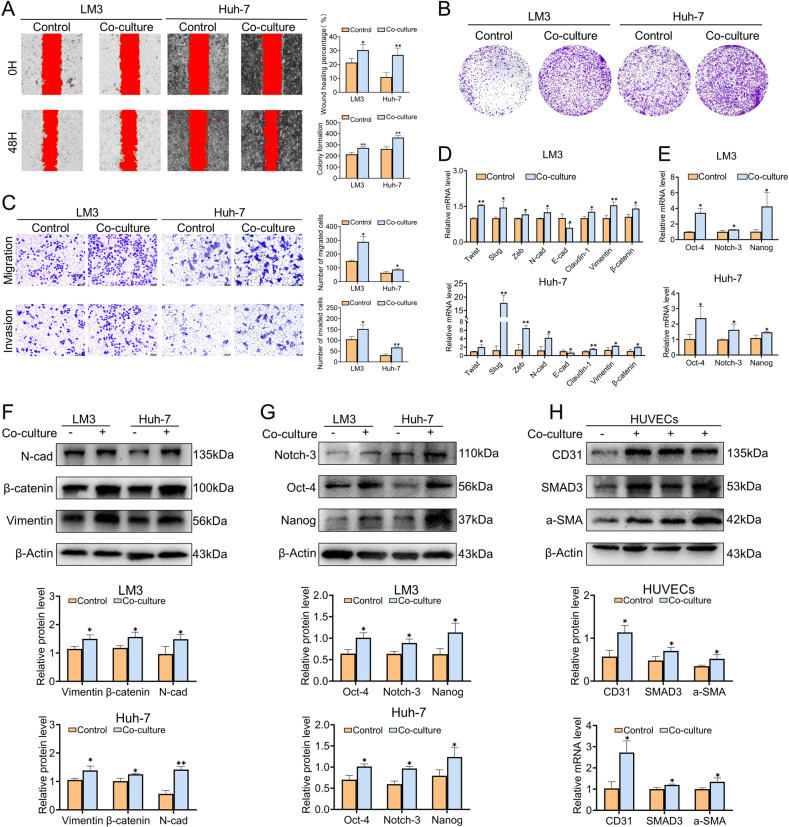
Th17 cells promote the HCC recurrence in vitro. **A** Cell mobility was analyzed using a wound-healing assay. **B** The effect of Th17 cells on the proliferative capacity of HCC cells. **C** The migratory and invasive abilities of HCC cells were detected by using Transwell assays. **D**, **F** The effect of Th17 cells on EMT indicators of HCC cells at mRNA (**D**) and protein (**F**). **E**, **G** The effect of Th17 cells on cancer stemness marker at mRNA (**E**) and protein (**G**) of HCC cells. **H** The promoting effect of Th17 cells on the angiogenesis of HUVECs. Changes of protein and mRNA of a-SMA, CD31, SMAD3 after co-culture with Th17 cells. Mean ± SEM, *p < 0.05, **p < 0.01, ***p < 0.001, Student *t*-test.

The expression trend of Th17 cells in liver IRI and the relationship between liver IRI and HCC recurrence have been clarified, so it is reasonable to assume that there might be a correlation between IRI-induced polarization of Th17 cells and HCC recurrence. The co-culture experiment of Th17 cells obtained by polarizing in vitro with HCC cells presented that, compared to the control group, HCC cells in the co-culture group were endowed with the superior ability of migration (Fig. 8A), proliferation (Fig. 8B), and invasion (Fig. 8C). Moreover, it was demonstrated that the expression of EMT indicators in the co-culture group was upregulated at the mRNA (Fig. 8D) and protein (Fig. 8F) levels, and the cancer stemness index including Nanog, Notch-3, and Oct-4 were also observed to be upregulated at mRNA (Fig. 8E) and protein level (Fig. 8G). In addition, the effect of Th17 cells in promoting angiogenesis was confirmed by the co-culture of Th17 cells with HUVECs (Fig. 8H). These results illuminated that, Th17 cells have the ability to promote HCC cell migration and invasion, enhance cancer stemness, activate the EMT program, and promote angiogenesis at the cellular level, which could be potential mechanisms to promote HCC recurrence.

**Fig. 9:**
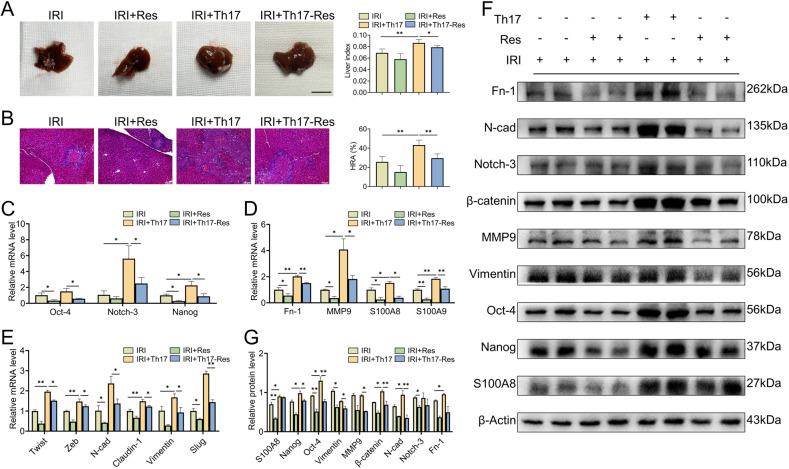
Th17 cells promote HCC recurrence in vivo. **A** Liver tissue images of mice with HCC recurrence and liver index (liver weight/body weight), *n* = 4–5, scale bars, 1 cm. **B** H&E staining to observe the morphology of recurrent tumors and hepatic replacement area (HRA) of HCC recurrence, *n* = 4–5. **C**, **D**, **F** The mRNA levels of cancer stemness indicators (**C**), pre-metastasis microenvironment indicators (**D**), and EMT indicators (**E**) in mice models. **F** Western blotting analysis of EMT indicators, cancer stemness indicators, and pre-metastasis microenvironment indicators in mice models. **G** Quantization of Western analysis. mean ± SEM, *p < 0.05, **p < 0.01, Student *t*-test.

Besides, the effect of Th17 cells in promoting HCC recurrence in vivo was observed by the adoptive transfer of polarized Th17 cells. Compared with the only sparse single tumor nodules in control group, the liver, after adoptively transferring of Th17 cells, presented large and multiple tumor nodules, indicating that Th17 cells have the property of promoting HCC recurrence, and up-regulation of FOXO1 in Th17 cells can partially attenuate the promoting effect (Fig. 9A, B). Moreover, it was indicated that in the HCC recurrence model, the cancer stemness pathway (Fig. 9C), the pre-metastasis microenvironment factor (Fig. 9D) and the EMT program (Fig. 9E) in vivo were activated at the mRNA level after adoptive transfer of Th17 cells, and the same trend was reflected at the protein level (Fig. 9F, G), while the function of FOXO1 in negatively regulating Th17 cells and stabilizing the liver microenvironment were confirmed in HCC recurrence models.

These results demonstrated that Th17 cells might have the ability to aggravate HCC recurrence by activating the EMT program, inducing cancer stemness, and promoting the formation of the pre-metastatic microenvironment and angiogenesis in vitro and in vivo, while the characteristics of FOXO1 in negatively regulating Th17 cells to reduce the HCC recurrence was recognized.

## Discussion

The high recurrence rate of HCC after hepatectomy has been an unresolved problem that seriously affects the prognosis of patients [33]. Liver IRI is a main complication of clinical liver surgery, such as hepatectomy and liver transplantation, and has always been the focus of clinical and basic research [34, 35]. Current research indicated that hepatic IRI is an independent precipitating factor for HCC recurrence, while the specific mechanism has not been thoroughly explored [5]. Based on previous research, this paper focused on the imbalance of immune homeostasis caused by ischemic stress, related hepatic IRI to HCC recurrence, and explored the potential mechanism of IRI-induced HCC recurrence.

Th17 cells are pro-inflammatory lymphocytes, which are widely recognized in autoimmune and inflammatory reactions [36]. Consistent with our study, in the acute inflammatory response of liver IRI, the polarization of Th17 cells was increased and involved in the outbreak of inflammation. Th17 cells and Treg cells are regarded as a pair of lymphocytes with opposite effects [16]. Treg cells have the ability to secrete anti-inflammatory factors such as IL-10, TGF-β, and IL-35 to inhibit the activation and proliferation of effector T cells, while Treg can also reduce the levels of IL-17 and IFN-γ and alleviate the progression of inflammation [37]. Current studies on Th17 cells in inflammatory response mostly focus on the balance of Th17/Treg cells. In previous studies of inflammatory diseases, researchers have tried to alleviate inflammation by targeting the regulation of Th17/Treg cell balance [26, 38]. Considering the homology between Th17 cells and Treg cells, which are differentiated from Naive CD4+ T cells under different polarization conditions, we extracted Naive CD4+ T cells from the mice IRI model for transcriptome sequencing and analysis. As a result, FOXO1 was screened as a key transcription factor involved in regulating DEGs of Naive CD4+ T cells under liver IR stress. And targeting FOXO1 confirmed that FOXO1 has the ability to inhibit Th17 cell polarization and increase Treg cell polarization in hepatic IRI.

Previous clinical observational studies have indicated that hepatic IRI is one of the risk factors for HCC recurrence, but the underlying mechanism has not been fully elucidated [6, 39]. Under normal conditions, IR stress activates the mechanisms of repairment and regeneration in the liver, initiates the differentiation of stem cells to support epithelial cells for resisting injury, and simultaneously initiates the EMT program to promote epithelial cell migration and repairmen [40]. However, in the context of residual HCC cells, the initiation of these programs in the liver probably serves as an incentive for HCC recurrence. Th17 cells and their cytokine IL-17A have been proven to promote the proliferation and invasion ability of tumor cells in cancer immunity, thus inducing cancer recurrence and metastasis [13, 41]. However, few studies have investigated the function of Th17 cells in HCC. In our study, we found that the expression of Th17 cells and their cytokines increased both in liver IRI and HCC recurrence models. Considering the tumor-promoting characteristics of Th17 cells, it is reasonable to assume that increased polarization of Th17 cells induced by liver IRI might be involved in the process of HCC recurrence. And our research results confirmed the role of Th17 cells in HCC recurrence for the first time, and indicated that Th17 cells could participate in the process of IRI-induced HCC recurrence in vitro and in vivo through inducing cancer stemness pathway and EMT program, remodeling the hepatic pre-metastatic microenvironment, and promoting angiogenesis. Therefore, based on our results, it seems that Th17 cells and their cytokines have the potential to be served as prognostic indicators, and their high infiltration abundance could be considered a risk factor for postoperative HCC recurrence.

FOXO1, as a class of indispensable transcriptional regulator in maintaining the formation and differentiation of immune cells, is normally expressed on Treg cells and participates in the network of functional regulation of Treg cells, and has the ability to recruit Treg cells and induce differentiation to achieve anti-inflammatory effects [20, 42, 43]. Nevertheless, FOXO1 has the inhibitory effect of forming a complex between FOXO1 and RORγt, thereby inhibiting the transcriptional activity of RORγt, which is the main transcription factor of Th17 cells [44–46]. And the role of FOXO1 in regulating the polarization of Th17 cells was first revealed in IRI-induced HCC recurrence. Our results supported that FOXO1 can alleviate the microenvironmental disturbance and reverse Th17/Treg cells imbalance, thus having a certain effect on relieving IRI-induced HCC recurrence. Besides, the function of FOXO1 and its correlation with Th17/Treg cells were identified. FOXO1 has function to attenuate the liver inflammatory response and the disturbance of immune microenvironment by negatively regulating the polarization and function of Th17 cells, thereby reducing the IRI-induced HCC recurrence. Therefore, FOXO1 has the potential to become a therapeutic target to regulate the polarization of Th17 cells and alleviate the HCC recurrence after surgery, providing a new research direction for subsequent basic and clinical studies.

## Conclusion

This paper systematically demonstrated the increased proportion of Th17 cells stimulated by hepatic IRI has the ability to reshape the liver microenvironment by activating the EMT program, and cancer stemness pathway, promoting the formation of pre-metastatic microenvironment and angiogenesis, making it more prone to the colonization of residual HCC cells and ultimately contribute to HCC recurrence. And FOXO1 has ability to stabilize the liver microenvironment and reduce HCC recurrence by inhibiting the polarization and function of Th17 cells, which may be used as a potential therapeutic target to alleviate IRI-induced HCC recurrence.

## Supplementary information

Reproducibility checklist

supplemental material

Original Data File

## Data Availability

The datasets generated during and/or analyzed during the study are available from the corresponding author upon reasonable request.
