# Supplementary material for: FOXO1 regulates Th17 cell-mediated hepatocellular carcinoma recurrence after hepatic ischemia-reperfusion injury
Source: Cell Death Dis. 2023 Jun 17;14(6):367. doi: 10.1038/s41419-023-05879-w (PMC10276824; doi:10.1038/s41419-023-05879-w)
Supplement: Supplementary file 2 — supplemental material [file 41419_2023_5879_MOESM2_ESM.docx]

**Table1. Antibodies for western blotting and immunohistochemical staining**

| **Antibody** | **Supplier** | **Catalog no.** | **Dilution** |
| --- | --- | --- | --- |
| β-Actin | Proteintech | 66009-1-Ig | 1:1000 |
| IL-17A | Invitrogen | PA5-79470 | 1:1000 |
| FOXP3 | Abcam | Ab215206 | 1:1000 |
| Fn-1 | Abcam | Ab268020 | 1:1000 |
| MMP9 | Abcam | Ab283575 | 1:1000 |
| FOXO1 | Abcam | Ab52857 | 1:1000 |
| S100A8 | Proteintech | 15792-1-AP | 1:1000 |
| Notch-3 | Proteintech | 55114-1-AP | 1:1000 |
| N-cadherin | Abcam | Ab76011 | 1:5000 |
| β-catenin | Proteintech | 17565-1-AP | 1:2000 |
| Vimentin | Proteintech | 10366-1-AP | 1:2000 |
| Oct-4 | Abcam | ab109183 | 1:1000 |
| Nanog | Abcam | Ab203919 | 1:1000 |
| CD31 | Proteintech | 28083-1-AP | 1:1000 |
| SMAD3 | Abcam | Ab40854 | 1:1000 |
| a-SMA | Proteintech | 14395-1-AP | 1:1000 |

**Table2. Primer sequences used in qPCR.**

| **Primer** | **species** | **Sequence (5' to 3')** | |
| --- | --- | --- | --- |
| β-Actin | Mouse | Forward | CAT CCG TAA AGA CCT CTA TGC CAA C |
|  |  | Reverse | ATG GAG CCA CCG ATC CAC A |
| Foxp3 | Mouse | Forward | ACC ATT GGT TTA CTC GCA TGT |
|  |  | Reverse | TCC ACT CGC ACA AAG CAC TT |
| IL-17A | Mouse | Forward | GGC CCT CAG ACT ACC TCA AC |
|  |  | Reverse | TCT CGA CCC TGA AAG TGA AGG |
| IL-17RA | Mouse | Forward | CGG AGA ATT AGT CCC TGT GTT G |
|  |  | Reverse | GAA CAG TCA CTT CAT ACT CCT GG |
| IL-6 | Mouse | Forward | TTG GGA CTG ATG CTG GTG AC |
|  |  | Reverse | GTG GTA TAG ACA GGT CTG TTG GG |
| IL-10 | Mouse | Forward | CTT ACT GAC TGG CAT GAG GAT CA |
|  |  | Reverse | GCA GCT CTA GGA GCA TGT GG |
| IL-1β | Mouse | Forward | AAG AAG AGC CCA TCC TCT GTG |
|  |  | Reverse | TGT TCA TCT CGG AGC CTG TAG |
| TNF-α | Mouse | Forward | CAG GCG GTG CCT ATG TCT C |
|  |  | Reverse | CGA TCA CCC CGA AGT TCA GTA G |
| Twist | Mouse | Forward | CTT GTG CCA AGT CTG GAG ATG |
|  |  | Reverse | ACG GTC AGC CTC TAG TGT TGT G |
| TGF-β | Mouse | Forward | CCA CCT GCA AGA CCA TCG AC |
|  |  | Reverse | CTG GCG AGC CTT AGT TTG GAC |
| β-Actin | Human | Forward | ATT GCC GAC AGG ATG CAG AA |
|  |  | Reverse | GCT GAT CCA CAT CTG CTG GAA |
| Foxp3 | Human | Forward | GTG GCC CGG ATG TGA GAA G |
|  |  | Reverse | GGA GCC CTT GTC GGA TGA TG |
| IL17A | Human | Forward | AGA TTA CTA CAA CCG ATC CAC CT |
|  |  | Reverse | GGG GAC AGA GTT CAT GTG GTA |
| IL17RA | Human | Forward | GCT TCA CCC TGT GGA ACG AAT |
|  |  | Reverse | TAT GTG CAT GTG CTC AAA |
| IL6 | Human | Forward | GTA GTG AGG AAC AAG CCA GAG C |
|  |  | Reverse | GTT GGG TCA GGG GTG GTT AT |
| IL10 | Human | Forward | GAC TTT AAG GGT TAC CTG GGT TG |
|  |  | Reverse | TCA CAT GCG CCT TGA TGT CTG |
| IL1β | Human | Forward | GAA TCT CCG ACC ACT ACA |
|  |  | Reverse | GCA CAT AAG CCT CGT TAT CCC |
| TNF-α | Human | Forward | CCT CTC TCT AAT CAG CCC TCT G |
|  |  | Reverse | GAG GAC CTG GGA GTA GAT GAG |
| Twist | Human | Forward | GTC CGC AGT CTT ACG AGG AG |
|  |  | Reverse | GCT TGA GGG TCT GAA TCT TGC T |
| TGF-β | Human | Forward  Reverse | GGC CAG ATC CTG TCC AAG C  GTG GGT TTC CAC CAT TAG CAC |
| FOXO1 | Mouse | Forward | CCC AGG CCG GAG TTT AAC C |
|  |  | Reverse | GTT GCT CAT AAA GTC GGT GCT |
| FOXO1 | Human | Forward | TCG TCA TAA TCT GTC CCT ACA CA |
| MMP9  Fn-1  S100A8  S100A9  Zeb  N-cadherin  Claudin-1  Vimentin  Slug  Oct-4  Notch-3  Nanog  Oct-4  Notch-3  Nanog  Slug  Zeb  N-cadherin  E-cadherin  Claudin-1  Vimentin  β-catenin  CD31  SMAD3  a-SMA | Mouse  Mouse  Mouse  Mouse  Mouse  Mouse  Mouse  Mouse  Mouse  Mouse  Mouse  Mouse  Human  Human  Human  Human  Human  Human  Human  Human  Human  Human  Human  Human  Human | Reverse  Forward  Reverse  Forward  Reverse  Forward  Reverse  Forward  Reverse  Forward  Reverse  Forward  Reverse  Forward  Reverse  Forward  Reverse  Forward  Reverse  Forward  Reverse  Forward  Reverse  Forward  Reverse  Forward  Reverse  Forward  Reverse  Forward  Reverse  Forward  Reverse  Forward  Reverse  Forward  Reverse  Forward  Reverse  Forward  Reverse  Forward  Reverse  Forward  Reverse  Forward  Reverse  Forward  Reverse  Forward  Reverse | CGG CTT CGG CTC TTA GCA AA  GCA GAG GCA TAC TTG TAC CG  TGA TGT TAT GAT GGT CCC ACT TG  ATG TGG ACC CCT CCT GAT AGT  GCC CAG TGA TTT CAG CAA AGG  AAA TCA CCA TGC CCT CTA CAA G  CCC ACT TTT ATC ACC ATC GCA A  ATA CTC TAG GAA GGA AGG ACA CC  TCC ATG ATG TCA TTT ATG AGG GC  ACC GCC GTC ATT TAT CCT GAG  CAT CTG GTG TTC CGT TTT CAT CA  AGG CTT CTG GTG AAA TTG CAT  GTC CAC CTT GAA ATC TGC TGG  TGC CCC AGT GGA AGA TTT ACT  CTT TGC GAA ACG CAG GAC AT  CGT CCA CAC GCA CCT ACA G  GGG GGA TGA GGA ATA GAG GCT  CAG CGA ACT GGA CAC ACA CA  ATA GGG CTG TAT GCT CCC GAG  AGA GGA TCA CCT TGG GGT ACA  CGA AGC GAC AGA TGG TGG TC  AGT GCC GAT CTG GTA CAA CTT  CAC TAC GGG GTT CTC ACA CA  CAC AGT TTG CCT AGT TCT GAG G  GCA AGA ATA GTT CTC GGG ATG AA  CTG GGT TGA TCC TCG GAC CT  CCA TCG GAG TTG CTC TCC A  TGG CGA CCT CAC TTA CGA CT  CAC TGG CAG TTA TAG GTG TTG AC  TTT GTG GGC CTG AAG AAA ACT  AGG GCT GTC CTG AAT AAG CAG  CGA ACT GGA CAC ACA TAC AGT G  CTG AGG ATC TCT GGT TGT GGT  GAT GAT GAA TGC GAG TCA GAT GC  ACA GCA GTG TCT TGT TGT TGT  TCA GGC GTC TGT AGA GGC TT  ATG CAC ATC CTT CGA TAA GAC TG  CGA GAG CTA CAC GTT CAC GG  GGG TGT CGA GGG AAA AAT AGG  CCT CCT GGG AGT GAT AGC AAT  GGC AAC TAA AAT AGC CAG ACC T  GAC GCC ATC AAC ACC GAG TT  CTT TGT CGT TGG TTA GCT GGT  AAAGCGGCTGTTAGTCACTGG  CGAGTCATTGCATACTGTCCAT  AAC AGT GTT GAC ATG AAG AGC C  TGT AAA ACA GCA CGT CAT CCT T  TGG ACG CAG GTT CTC CAA AC  CCG GCT CGC AGT AGG TAA C  GGC ATT CAC GAG ACC ACC TAC  CGA CAT GAC GTT GTT GGC ATA C |
