# Supplementary material for: FOXO1 regulates Th17 cell-mediated hepatocellular carcinoma recurrence after hepatic ischemia-reperfusion injury
Source: Cell Death Dis. 2023 Jun 17;14(6):367. doi: 10.1038/s41419-023-05879-w (PMC10276824; doi:10.1038/s41419-023-05879-w)
Supplement: Supplementary file 3 — Original Data File [file 41419_2023_5879_MOESM3_ESM.pptx]

## Slide 1
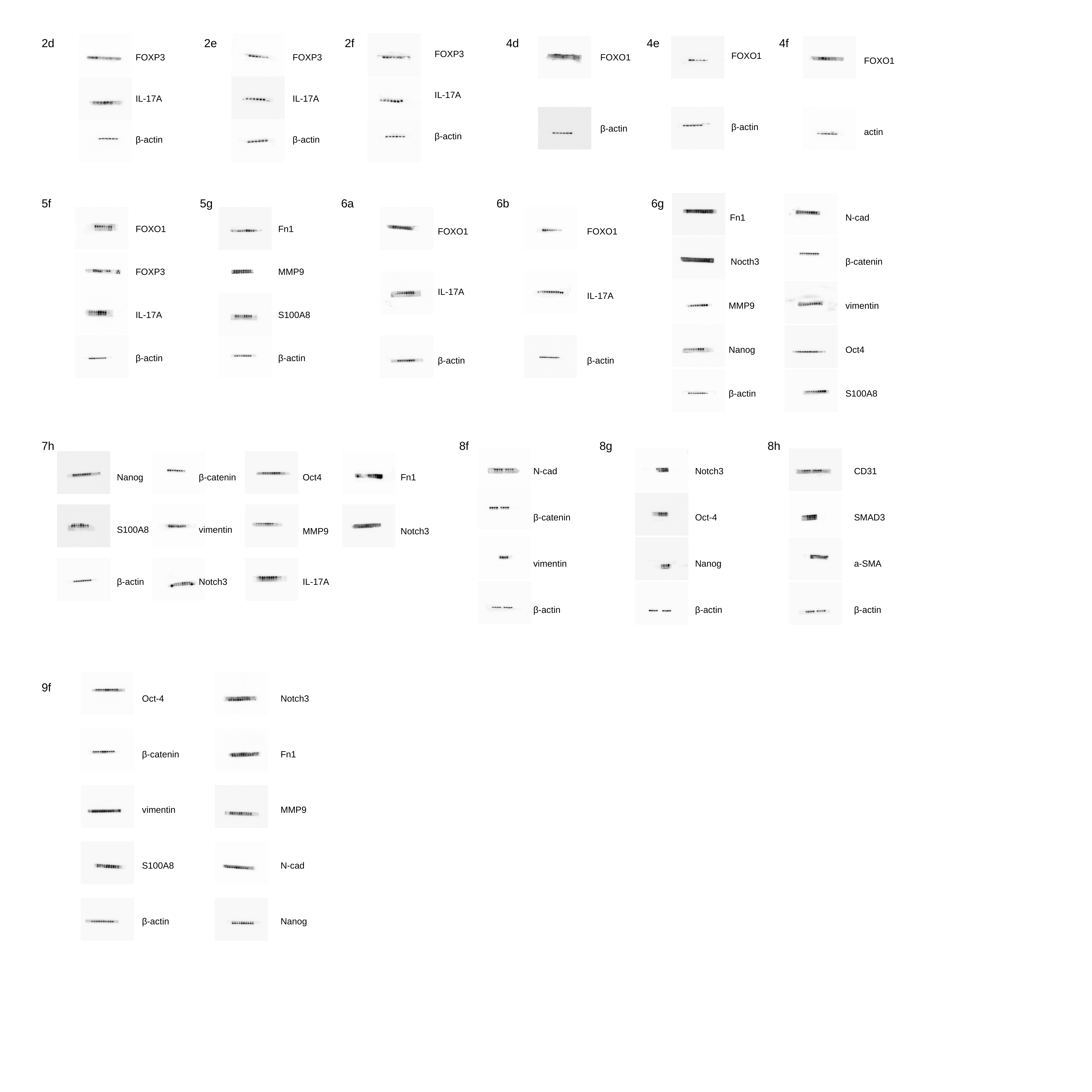

2d
2e
2f
4d
4e
4f
FOXP3
FOXO1
FOXO1
FOXP3
FOXP3
FOXO1
IL-17A
IL-17A
IL-17A
β-actin
β-actin
actin
β-actin
β-actin
β-actin
5f
5g
6a
6b
6g
Fn1
N-cad
FOXO1
Fn1
FOXO1
FOXO1
Nocth3
β-catenin
FOXP3
MMP9
IL-17A
IL-17A
MMP9
vimentin
IL-17A
S100A8
Nanog
Oct4
β-actin
β-actin
β-actin
β-actin
β-actin
S100A8
7h
8f
8g
8h
N-cad
Notch3
CD31
Nanog
β-catenin
Oct4
Fn1
β-catenin
Oct-4
SMAD3
vimentin
S100A8
MMP9
Notch3
vimentin
Nanog
a-SMA
β-actin
Notch3
IL-17A
β-actin
β-actin
β-actin
9f
Oct-4
Notch3
β-catenin
Fn1
vimentin
MMP9
S100A8
N-cad
β-actin
Nanog
